# Genomic variability in *Potato virus M *and the development of RT-PCR and RFLP procedures for the detection of this virus in seed potatoes

**DOI:** 10.1186/1743-422X-7-25

**Published:** 2010-02-01

**Authors:** Huimin Xu, Jeanette D'Aubin, Jingbai Nie

**Affiliations:** 1 Canadian Food Inspection Agency, Charlottetown Laboratory, 93 Mount Edward Road, Charlottetown, PEI, C1A 5T1, Canada

## Abstract

*Potato virus M *(PVM, *Carlavirus*) is considered to be one of the most common potato viruses distributed worldwide. Sequences of the coat protein (CP) gene of several Canadian PVM isolates were determined. Phylogenetic analysis indicated that all known PVM isolates fell into two distinct groups and the isolates from Canada and the US clustered in the same group. The Canadian PVM isolates could be further divided into two sub-groups. Two molecular procedures, reverse transcription - polymerase chain reaction (RT-PCR) and restriction fragment length polymorphism (RFLP) were developed in this study for the detection and identification of PVM in potato tubers. RT-PCR was highly specific and only amplified PVM RNA from potato samples. PVM RNAs were easily detected in composite samples of 400 to 800 potato leaves or 200 to 400 dormant tubers. Restriction analysis of PCR amplicons with *Msc*I was a simple method for the confirmation of PCR tests. Thus, RT-PCR followed by RFLP analysis may be a useful approach for screening potato samples on a large scale for the presence of PVM.

## Background

*Potato virus M *(PVM), a member of the genus *Carlavirus *in the family *Flexviridae*, has a single-stranded, polyadenylated, positive-sense genomic RNA of appropriately 8.5 kb in length [1,2]. PVM is considered to be one of the most common potato viruses distributed worldwide and an economically important pathogen of potato (*Solanum tunerosum*). PVM can cause a yield reduction in potatoes between 15% and 45%, and potato cultivars may be 100% infected in some regions [3]. The virus is transmitted by aphids in a non- persistent manner and by mechanical inoculation with sap from young leaves [1]. PVM causes mottle, mosaic, crinkling and rolling of leaves and stunting of shoots. Symptoms of potato plants caused by PVM infection are similar to those caused by several other common potato viruses including *Potato virus S *(PVS, Carlavirus), *Potato virus X *(PVX, *Potexvirus*) and the common strain of *Potato virus Y *(PVY^O^, *Potyvirus*). Severity of symptoms varies greatly depending on the combination of potato cultivars and PVM isolates [3,4].

A practical and important way to limit the spread of PVM and to control potato disease caused by this virus is to use PVM-free potato seed tubers. It is required by seed potato certification program in Canada and many other countries that seed potatoes must be screened for various viruses including PVM and the total virus incidence must be lower than an acceptable level (*e.g*. 5%). Currently enzyme-linked immunosorbent assay (ELISA) is the predominant method employed for the detection of PVM in potato samples on a large scale [5,6]. But, to screen potato tuber samples by ELISA, the tuber dormancy must be broken and sprouts are used for detecting PVM to avoid false negative result due to the low PMV titre in dormant potato tubers. Reverse transcription - polymerase chain reaction (RT-PCR) procedures have been developed and employed successfully for the specific detection of several potato viruses including various strain groups of PVY [6-11], *Potato mop-top virus *(PMTV, *Pomovirus*)[12], *Tobacco rattle virus *(TRV, *Tobravirus*)[13] and *Alfalfa mosaic virus *(AMV, *Alfamovirus*)[14]. RT-PCR has been demonstrated to be sensitive, specific, simple and fast. The efficiency of viral or total RNA extraction from potato samples on a large scale has been greatly improved by the utilization of standard commercial RNA extraction kits [13,14]. Viral RNA can be extracted directly from dormant potato tubers without the need to treat the tubers for breaking dormancy or to grow the tubers in greenhouse for leaf testing by ELISA.

In this paper we report on the analysis of the coat protein (CP) gene sequence of several Canadian PVM isolates and the comparison of PVM isolates from Canada and other countries. Oligonucleotide primers specific to PVM CP gene were designed and RT-PCR procedures were developed for the specific detection of PVM in various potato samples and for the confirmation of PCR amplicons. The efficacy of RT-PCR for indexing seed potato samples on large scale for PVM was enhanced by using composite leaf and tuber samples. Restriction fragment length polymorphism (RFLP) was introduced to verify PCR amplicon identity.

## Results

### Initial tests by ELISA and RT-PCR

**Figure 1:**
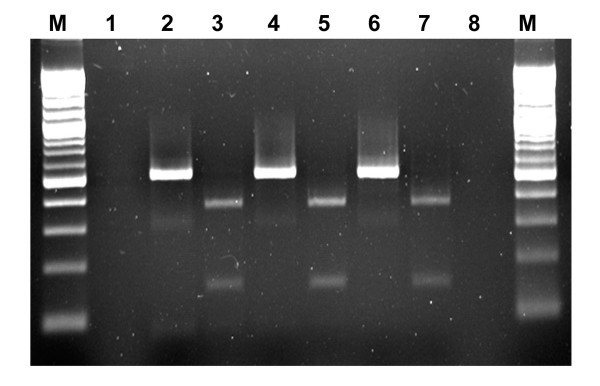
**Detection of PVM RNA in potato samples by RT-PCR and confirmation of PCR products by restriction analysis**. PCR amplicons (520 bp) were produced in RT-PCR using primers PVM3 and PVM4 from RNA extracted from PVM-Ca508 infected tuber (lanes 2), leaf (lane 4) and sprout (lanes 6) samples. PCR products were digested into two fragments, 370 and 150 bp with *Msc*I (lanes 3, 5, 7) for verification. M: Molecular weight marker (100 bp DNA ladder, New England Biolabs, Pickering, Ontario); No-template control (lane 1) and negative control (RNA extracted from healthy leaves, lane 8) were used for PCR. Gel electrophoresis: 1.5% Agarose-1000 (Invitrogen Canada, Burlington, Ontario).

**Figure 2:**
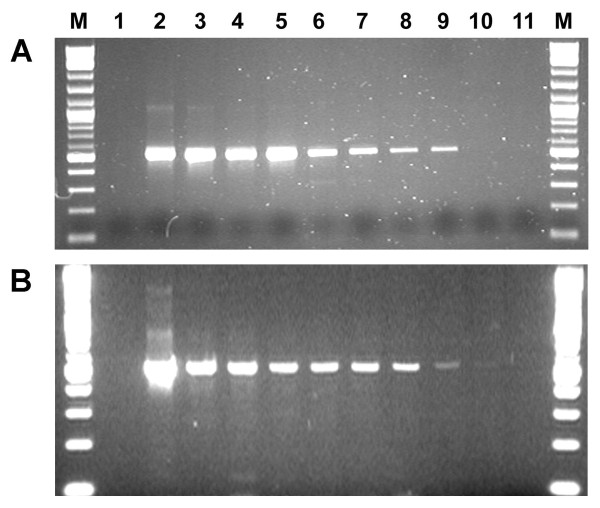
**Sensitivity of RT-PCR using primer set PVM3/PVM4 for detecting PVM in composite sprout (A) and dormant tuber (B) samples**. RNA extracted from mixtures of infected (with PVM isolate Ca508) and healthy sprout or tuber sap at ratios of 1:0, 1:4, 1:9, 1:24, 1:49, 1:99, 1:199, 1:399, 1:799 and 0:1 (lanes 2-11, respectively). PCR amplicons: 520 bp. M: Molecular weight marker (100 bp DNA ladder, New England Biolabs, Pickering, Ontario); Lane 1: no-template control for PCR.

All original PVM samples (tubers, leaves, tissue culture plantlets) were confirmed by ELISA to be positive for PVM (Table 1). In preliminary tests, amplicons of 520 bp were generated in RT-PCR using primer set PVM3/PVM4 from RNA templates extracted from leaf samples of potato plants infected with PVM isolates CL1, 2, 3, 4 and several field isolates and all PCR amplicons were digested by *Msc*I specifically resulting in two fragments of expected size (Table 1, Fig. 1). RNA extracts from foliage of potato plants infected with other viruses or viroid were also tested in RT-PCR using this primer set to determine specificity. None of them yielded any amplification products. To evaluate the sensitivity of the RT-PCR for the detection of PVM in dormant tubers using primers PVM3/PVM4, sap from PVM infected tubers was mixed with sap from healthy tubers in a dilution series from 1:0 to 1:799 (infected tissue sap: healthy tissue sap, v/v) followed by RNA extraction. PCR amplicons of 520 bp were detected in all composite leaf samples (from 1:0 to 1:799, data not shown) and in most of the composite tuber (dormant) and sprout samples ranging from 1:0 to 1:399 (Fig. 2).

**Table 1:** Identification of PVM isolates in potato samples*

Isolates	Potato Cultivar	Origin	Symptoms	ELISA^a ^Test 1/Test 2	PCR/RFLP^b^
Isolates					
CL1	Shepody	OLF	Mosaic	+/+	+/+
CL2	RB	OLF	Mosaic	+/+	+/+
CL3	GM	OLF	Mosaic	+/+	+/+
CL4	A82705-1	UI	Mosaic	+/+	+/+
Ca5	Shepody	PEI	Mosaic	+/+	+/+
Ca99	Shepody	PEI	Mosaic	+/+	+/+
Ca102	Shepody	PEI	Mosaic	+/+	+/+
Ca128	Shepody	PEI	Mosaic	+/+	+/+
Ca414	Shepody	PEI	Mosaic	+/+	+/+
Ca508	Shepody	PEI	Mosaic	+/+	+/+
Ca513	Shepody	PEI	Mosaic	+/+	+/+
					
Controls					
Negative	Shepody	CL	Healthy	-/-	-/-
NTC	N/A	N/A	N/A	-/-	-/-

*CL: Canadian Food Inspection Agency (CFIA) - Charlottetown Laboratory; OLF: CFIA - Ottawa Laboratory (Fellow Field); UI: University of Idaho (Idaho, USA); PEI: the province of Prince Edward Island; RB: Russet Burbank; GM: Green Mountain; NTC: no template control; N/A: not applicable; +: positive results; -: negative results.

a. The results are shown as initial test of samples/confirmatory test of inoculated plants (positive threshold: 0.1).

b. The results are shown as PCR amplification/RFLP confirmation using *Msc*I.

### Confirmatory tests

All samples confirmed to be positive for PVM in the initial RT-PCR tests were re-tested to confirm the validity of the initial test and as a check for false positive results. RNA was re-extracted from fresh tissue sap and amplified by RT-PCR using the same primer set (PVM3/PVM4). In each case RT-PCR amplicons of 520 bp were obtained and digested into 150 and 370 bp fragments upon treatment with *Msc*I (Table 1, Fig. 1). Retested samples that gave the expected RT-PCR and RFLP results were considered confirmed positives. Tissue saps of all positive samples were used to inoculate potato and indicator plants. All these test PVM isolates induced light mosaic symptoms on inoculated potato plants (Shepody) (Table 1) and chlorotic local lesions on inoculated *Chenopodium quinoa *plants (data not shown). Subsequently PVM was detected by ELISA, RT-PCR and RFLP (the same as that shown in Table 1) from inoculated plants. Progeny tubers produced by the inoculated plants did not show any necrotic symptom (neither surface, nor internal) at the time of harvest or after 12 weeks in storage at 4-8°C.

### Sequence analysis

**Figure 3:**
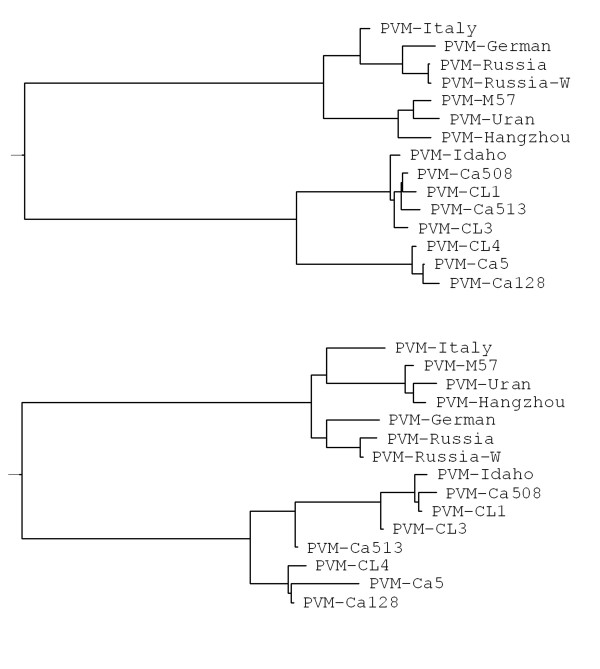
**Phylogenetic dendrogram depicting the relationship among PVM isolates based on alignment of nucleotide of the CP gene (top) and amino acid sequence of the coat protein (bottom)**. The phylogenetic relationship between PVM isolates were deduced using the Bootstrap Neighbour-Joining (N-J) methods (random number generator seed: 111, number of bootstrap trails: 1000) in the Phylip formatted Clustal W (V1.82). The trees were visualized and the dendrograms were displayed using the program TreeView (V1.5).

Total RNAs extracted from potato leaves confirmed to be positive for PVM by various tests (ELISA, bioassay, RT-PCR and RFLP) were also amplified in RT-PCR using primer set PVM1/PVM2 flanking the entire CP gene of PVM resulting in PCR amplicons of 917 bp (CP gene: 915 bases) that were then sequenced from both directions using primers PVM1/PVM2. PCR amplicons generated using primer set PVM3/PVM4 were also sequenced from both directions with the same primer set. Sequence alignment and phylogenetic analysis based on the nucleotide sequence of the CP gene and the amino acid sequence of coat protein showed that all PVM isolates fell into two distinct groups - I and II (Table 2 and Fig. 3). PVM isolates in group I only shared approximately 73% - 75% of identical nucleotides and 85% - 87% of identical amino acids with isolates in group II. Isolates within the same group (either I or II) shared over 90% of identical nucleotides and over 95% identical amino acids (Table 2). Variation among isolates in either group (I or II) was 7% to 8% in nucleotides and approximately 4% in amino acids. PVM isolates in either group I or II might be further divided into two or three sub-groups (Table 2, Fig. 3).

**Table 2:** Comparison of nucleotide and amino acid sequences of the coat protein of PVM isolates from different countries*

PVM strain/isolate	PVM- Hangzhou	PVM- Italy	PVM- Russia	PVM- Idaho	PVM- Ca128	Reference
Hangzhou	-/-	93/96	94/96	75/87	73/85	AJ437481
M57	97/99	94/97	94/97	74/87	73/85	AY311395
Uran	97/99	94/96	94/96	74/86	73/85	AY311394
						
German	93/96	97/97	98/98	75/87	74/86	X57440
Italy	93/96	-/-	97/97	75/87	75/86	X85114
Russia	94/96	97/97	-/-	75/88	74/86	[2]
Russia-W	94/96	97/97	99/99	75/87	74/86	D14449
						
Idaho	75/87	75/87	75/88	-/-	92/96	AF023877
Ca508	74/87	75/87	75/88	99/99	92/96	EF063388
CL1	74/86	75/87	75/87	98/99	91/96	EF063383
CL3	75/86	75/87	75/87	99/99	91/96	EF063384
Ca513	74/85	75/86	75/86	98/98	91/95	EF063389
						
CL4	74/87	75/87	75/88	92/98	99/98	EF063385
Ca5	73/86	75/87	75/88	92/98	99/98	EF063386
Ca128	73/85	75/86	74/86	92/96	-/-	EF063387

* The length of the targeted region is 915 bases or 305 amino acids. The identity between two isolates or strains is shown as the percentage of identical bases/amino acids.

## Discussion

In this study, PVM was detected in several potato samples from an experimental farm in the province of Prince Edward Island (PEI), Canada. These PVM isolates and four other PVM isolates from Charlottetown Laboratory (CL), Canadian Food Inspection Agency (CFIA) virus collection were characterized in this study by ELISA, bioassay, RT-PCR, RFLP and sequence analysis of the CP gene. Data from all the tests positively identified the virus as PVM. Sequence analysis of these PVM isolates and eight other known PVM strains/isolates showed that all PVM isolates evaluated fell into two distinct groups - group I and II. Group I consisted of PVM isolates detected and characterized in Italy, Germany, China, Poland and Russia and group II consisted of Canadian and US isolates. Isolates in group I might be further divided into two sub-groups - Ia, and Ib. PVM isolates from China and Poland formed the group Ia and group Ib consisted of isolates from Italy, Germany and Russia. Isolates in group II could be further divided into two sub-groups - IIa and IIb. PVM isolates Idaho and Ca128 were considered to represent IIa and IIb, respectively. Group IIa consisted of isolates Idaho, Ca508, Ca513, CL1 and CL3 and group IIb consisted of CL4, Ca5 and Ca218 (Table 2, Fig. 3). PVM Idaho strain and PVM isolates detected in potato samples from PEI probably have the same origin and both group IIa and IIb types of PVM isolates may be present in the same geographic region.

Based on the sequence alignment of known PVM strains/isolates, two sets of primers specific to PVM CP gene, PVM1/PVM2 and PVM3/PVM4 were designed and were evaluated in RT-PCR for the detection of PVM in potato tubers (dormant and non-dormant), sprouts and leaves (also tissue culture plantlets). Both sets of primers were highly specific and only amplified PVM RNA to generate PCR products of the expected length. These primer sets produced no amplicons from potato samples collected from healthy plants and plants infected with *Potato spindle tuber viroid *(PSTVd, the type species of *Pospiviroid*) and several other viruses (see below). Primer set PVM1/PVM2 was used successfully for the amplification of the entire PVM CP gene. Primer set PVM3/PVM4 was employed mainly in RT-PCR for amplifying a segment of PVM CP gene from total RNAs extracted from various types of potato samples. PVM RNA was readily detected by RT-PCR from total RNA preparations extracted from bulked potato samples (leaves, sprouts and tubers). Up to 800 leaves or 400 sprouts could be combined for reliable detection of PVM RNA by RT-PCR. Up to 200 to 400 dormant tubers could be combined to achieve reliable detection of PVM RNA by RT-PCR. The approach of using composite tuber samples will greatly reduce the cost and time associated with RT-PCR for indexing seed potato lots on a large scale for the presence or absence of PVM. If there is need, it is possible to determine the quantity of PVM RNA in the test sample by real-time quantitative RT-PCR. In this study, PVM RNA was easily detectable from as low as 5 ng of total RNAs extracted from dormant potato tubers or as low as 0.1 pg of PVM RNA was readily detectable by real-time quantitative RT-PCR (data not shown).

Analysis of PCR amplicons based solely on molecular mass on agarose gels may result in false positive conclusions. RFLP analysis was conducted in this study to determine the identity of all PCR amplicons and the results confirmed that all amplicons generated by primers PVM1/PVM2 and PVM3/PVM4 were indeed derived from PVM RNAs. A single *Msc*I (TGG↓CCA) site was confirmed in the region flanked by primer PVM3 and PVM4 in the CP gene of all the Canadian PVM isolates sequenced in this study and all other known PVM isolates except those PVM strains/isolates (M57, Uran and Hangzhou) in the phylogenetic group Ia. RT-PCR using primer set PVM3/PVM4 followed by RFLP analysis using *Msc*I provided a rapid, sensitive and reliable detection-confirmation approach for indexing seed potatoes in Canada since all PVM samples from Canadian potato lots have been detected and identified using this approach. Several other restriction endonucleases, such as *Nco*I, *Nde*I, *Pvu*II and *Taq*I, were also evaluated and they may be used to differentiate various PVM strains/isolate since not all analysed PVM isolates have the restriction sites. For example, in the region between primers PVM3 and PVM4, a *NcoI *site was revealed in PVM isolates detected in Germany, Italy, Russia, Poland and China, but not found in the isolates detected in the US (Idaho strain) and Canada (data not shown).

## Conclusion

PVM isolates were characterized at the molecular level in this study and their genetic relationships with other known PVM isolates were established. RT-PCR and RFLP procedures were developed for the detection and identification of PVM in potatoes. Composite leaf or tuber samples can be used in PCR tests to reduce the time and cost needed for screening tubers on a large scale.

## Materials and methods

### Potato samples

Seven PVM isolates (Ca5, Ca99, Ca102, Ca128, Ca414, Ca508 and Ca513) from potatoes (*Solanum tuberosum *cv. Shepody) grown on an experimental farm in the province Prince Edward Island, Canada and four PVM isolates (CL1, 2, 3, 4) from CFIA-CL virus collection, were identified and characterized, on the basis of bioassay and serological reactions (Table 1). All isolates were maintained in infected potato plants that were grown under greenhouse conditions and progeny tubers were harvested and stored at 4°C. Dormancy breaking was done by maintaining tubers at room temperature. Tubers were sampled according to the methods described previously [12]. Leaf and sprout samples were directly used for extracting tissue sap. All samples were macerated in extraction bags (Bioreba, Reinach, Switzerland) by pounding the tissue with a hammer and the tissue sap was subjected to different tests as described below. Plant sap was kept at -20°C if it was not used within 12 hours. A second aliquot of the sap from RT-PCR positive samples was re-tested to confirm the initial results. All PCR amplicons were subjected to restriction digestion and the RFLP pattern was used to determine whether they matched PVM patterns. Positive samples were then subjected to bioassay (see below).

### Bioassay

Tissue sap from PVM positive samples based on initial ELISA and RT-PCR tests were inoculated onto healthy potato (Russet Burbank) and indicator (*C. quinoa*) plants. Tissue sap was diluted (1:2) in phosphate buffer (0.1 M, pH7.2) and inoculated onto leaves of potato and indicator plants by rubbing carborundum dusted leaves. Inoculated plants were grown in a greenhouse at 18°C - 22°C and observed every other day for the development of symptoms. All inoculated plants (regardless of whether or not symptoms developed) were tested for PVM by ELISA and RT-PCR. All progeny tubers were tested by RT-PCR followed by RFLP analysis for confirmation.

### ELISA

Potato tubers and leaves as well as indicator plants were screened for the presence of PVM by a standard double antibody sandwich ELISA using commercial coating antibody, conjugate, positive control and all necessary reagents from Neogen Europe Ltd. (Auchincruive, Scotland, UK), following the procedures recommended by the supplier. Tuber or leaf tissues were homogenized in 0.1 M phosphate buffer containing 0.02% NaN_3_, 0.1% Tween 20 and 0.1% skim milk powder (pH 7.4) at a sample to buffer ratio of 1:5 (w:v) and 100 μl of extracted sap was loaded in duplicate on microtitre plates. A panel of positive, negative, and buffer controls, in addition to the controls supplied with the ELISA kit, were included on each plate. Absorbance values (A_405 nm_) of 4 times of the healthy control reading was used as the positive threshold, but if absorbance of the healthy control was < 0.030, a positive threshold of 0.100 was used.

### RT-PCR

From each macerated sample, 100 μl of sap was extracted with the Tri-Reagent (Molecular Research Center, Inc, Cincinnati. OH) as described by the manufacturer. Subsequently the RNA was extracted with chloroform, precipitated with isopropanol, washed with ethanol and suspended in 25 μl (for tuber samples) or 50 μl (for sprouts, leaves and tissue culture plantlets) of RNase-free and DNase-free water according to the procedures described previously [12]. RNA extracts from leaves and tubers of healthy potato plants were used as negative controls. RNA extracts from foliage and/or tubers of potato plants infected with other viruses including AMV, PMTV, TRV, PVS, PVX, *Potato aucuba mosaic virus *(PAMV, *Potexvirus*), PVY^O^, the tobacco vein necrotic strain of PVY (PVY^N^), the potato tuber necrotic strain of PVY (PVY^NTN^), *Potato virus A *(PVA, *Potyvirus*), *Potato latent virus *(PotLV, *Carlavirus*), *Potato leafroll virus *(PLRV, *Polerovirus*) and PSTVd were also included in RT-PCR tests to determine primer specificity.

Two sets of primers were designed based on sequence analysis of known PVM isolates obtained from the NCBI website including the isolates Hangzhou (China, AJ437481), M57 (Poland, AY692075), Uran (Poland, AY311394), German isolate (X57440), Italy tomato strain (X85114), Idaho strain [15](USA, AF023877) and two Russian PVM isolates [2](D14449, Table 2). Primers PVM1 (Reverse: CTTCATTTGTTATTCGACTT) and PVM2 (Forward: ATGGGAGATTCAAC**R**AAGAA) were used for amplifying the entire CP gene and the nucleotide sequences of the amplicons (917 bp) were then determined in both directions using the same primer set. PVM3 (Reverse: TGAGCTCGGGACCATTCATAC) and PVM4 (Forward: ACATCTGAGGACATGATGCGC) were used in RT-PCR and real-time RT-PCR to yield an amplicon of 520 bp.

First strand cDNA synthesis was carried out using Moloney murine leukemia virus (M-MLV) reverse transcriptase (Invitrogen Canada, Burlington, ON, Canada) using the antisense primer (PVM1 or PVM3). The procedure for the two step RT-PCR were essentially the same as described previously [11]. A reaction containing no cDNA template was included in all PCR tests as a blank control. The temperature regime for amplification reactions was as follows: initial denaturation for 5 min at 95°C, followed by 35 cycles of 94°C for 45 seconds, 58°C for 45 seconds, and 72°C for 45 seconds. The final extension was at 72°C for 7 min. A GeneAmp 9700 thermocycler (Applied Biosystems, Foster City, CA) was used for RT-PCR amplifications. Optimal annealing temperature of primers was determined using a temperature gradient thermocycler (Watman Biometra, Goettingen, Germany). PCR products were separated on a 1.2% agarose gel, stained with ethidium bromide, and visualized under UV light.

### Restriction digestion

Several restriction endonucleases *Msc*I, *Nde*I, *Pst*I, *Pvu*II and *Taq*I (New England Biolabs, Pickering, ON, Canada) were evaluated for a direct digestion of PCR amplicons generated with primers PVM3/PVM4 and *Msc*I was selected for all RFLP tests in this study. Five microliters of PCR amplicons were digested with 5 units of the restriction enzyme at 37°C for 1 hour in a reaction volume of 20 μl using the buffer recommended by the enzyme supplier. RFLP patterns were analysed by agarose gel electrophoresis using 1.5% agarose-1000 (Invitrogen Canada, Burlington, ON, Canada) stained with ethidium bromide and visualized under UV light.

### Sequence analysis

The CP gene amplicons generated in RT-PCR from test samples using primer PVM1/2 and PVM3/4 were purified using QIAquick PCR purification kit (Qiagne Inc., Mississauga, ON, Canada) following the procedures recommended by the provider and the dsDNAs amplified in a typical RT-PCR reaction (50 μl) were eluted with 30 μl of water (DNase-free and RNase-free). Purified PCR amplicons from the CP gene of PVM isolates CL1, CL3, CL4, Ca5, Ca128, Ca508 and Ca513 were sequenced in both orientations by automated cycle sequencing (York University, Toronto, ON, Canada) using primers PVM1/PVM2 and/or PVM3/PVM4 depending on the templates. Sequences of Canadian PVM isolates were compared with PVM sequences in the NCBI database with the program BLAST. Nucleotide and amino acid sequences were aligned using Clustal G (V1.1) and GeneDoc Multiple Sequence Alignment Editor and Shading Utility (V2.5.000)[16]. The phylogenetic relationship of the Canadian potato isolates of PVM and 8 other known PVM strains/isolates (Table 2) were deduced using the Bootstrap Neighbour-Joining (N-J) methods (random number generator seed: 111, number of bootstrap trails: 1000) in the Phylip formatted Clustal W (V1.82). The trees were visualized and the dendrograms were displayed using the program TreeView (V1.5).

## Competing interests

The authors declare that they have no competing interests.

## Authors' contributions

JD carried out the serological and biological characterizations. JN carried out the molecular characterizations of PVM isolates. HX designed and coordinated the study and carried out the genetic analysis. All authors read and approved the final manuscript.
